# Tirzepatide prevents neurodegeneration through multiple molecular pathways

**DOI:** 10.1186/s12967-024-04927-z

**Published:** 2024-01-29

**Authors:** Rosaria Anna Fontanella, Puja Ghosh, Ada Pesapane, Fatemeh Taktaz, Armando Puocci, Martina Franzese, Maria Federica Feliciano, Giovanni Tortorella, Lucia Scisciola, Eduardo Sommella, Concetta Ambrosino, Giuseppe Paolisso, Michelangela Barbieri

**Affiliations:** 1 Department of Advanced Medical and Surgical Sciences, University of Campania “Luigi Vanvitelli”, Naples, Italy; 2 Department of Pharmacy, University of Salerno, Fisciano, SA Italy; 3 Biogem Institute of Molecular Biology and Genetics, Ariano Irpino, Italy; 4 Department of Science and Technology, University of Sannio, Benevento, Italy; 5 UniCamillus, International Medical University, Rome, Italy

**Keywords:** Neurodegeneration, Diabetes mellitus type 2, Neuronal growth, Neurodifferentiation, Insulin resistance, Glucose homeostasis

## Abstract

**Background:**

Several evidence demonstrated that glucagon-like peptide 1 receptor agonists (GLP1-RAs) reduce the risk of dementia in type 2 diabetes patients by improving memory, learning, and overcoming cognitive impairment. In this study, we elucidated the molecular processes underlying the protective effect of Tirzepatide (TIR), a dual glucose-dependent insulinotropic polypeptide receptor agonist (GIP-RA)/ GLP-1RA, against learning and memory disorders.

**Methods:**

We investigated the effects of TIR on markers of neuronal growth (CREB and BDNF), apoptosis (BAX/Bcl2 ratio) differentiation (pAkt, MAP2, GAP43, and AGBL4), and insulin resistance (GLUT1, GLUT4, GLUT3 and SORBS1) in a neuroblastoma cell line (SHSY5Y) exposed to normal and high glucose concentration. The potential role on DNA methylation of genes involved in neuroprotection and epigenetic modulators of neuronal growth (miRNA 34a), apoptosis (miRNA 212), and differentiation (miRNA 29c) was also investigated. The cell proliferation was detected by measuring Ki-67 through flow cytometry. The data were analysed by SPSS IBM Version 23 or GraphPad Prism 7.0 software and expressed as the means ± SEM. Differences between the mean values were considered significant at a p-value of < 0.05. GraphPad Prism software was used for drawing figures.

**Results:**

For the first time, it was highlighted: (a) the role of TIR in the activation of the pAkt/CREB/BDNF pathway and the downstream signaling cascade; (b) TIR efficacy in neuroprotection; (c) TIR counteracting of hyperglycemia and insulin resistance-related effects at the neuronal level.

**Conclusions:**

We demonstrated that TIR can ameliorate high glucose-induced neurodegeneration and overcome neuronal insulin resistance. Thus, this study provides new insight into the potential role of TIR in improving diabetes-related neuropathy.

**Graphical Abstract:**

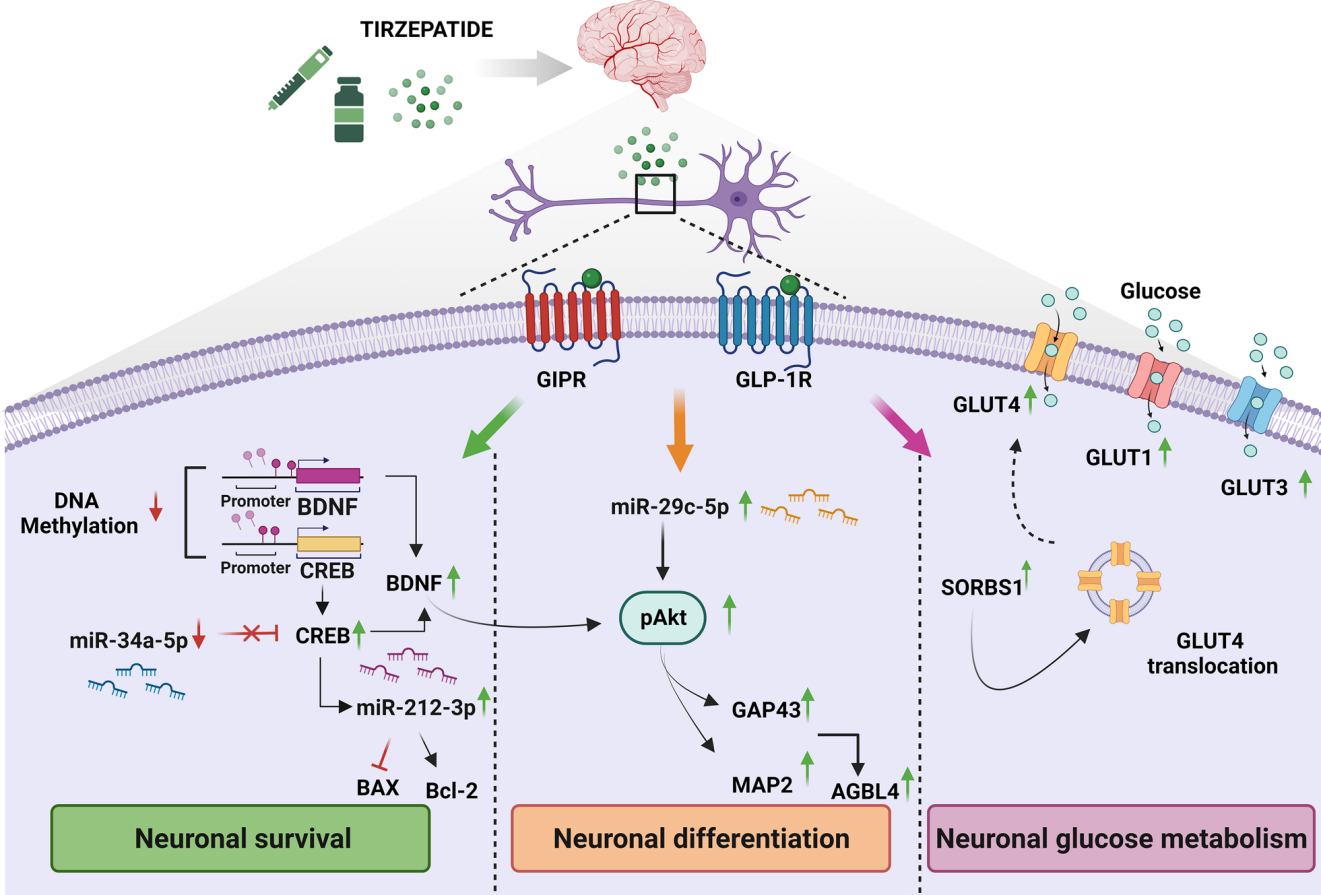

**Supplementary Information:**

The online version contains supplementary material available at 10.1186/s12967-024-04927-z.

## Background

Alzheimer's disease (AD) is multi-factorial, with genetic and environmental factors implicated in its pathogenesis [1]. The best-described risk factors for AD are age, a positive family history of dementia, low levels of education, female gender, previous depression, and vascular factors. Interestingly, large epidemiological evidence suggests that Type 2 Diabetes Mellitus (T2DM) is strongly associated with cognitive impairment and dementia due to failure in the action of glucose handling in the neurons as a consequence of impaired brain insulin signaling [2–4]. The deregulated availability of glucose causes a decrease in the rate of energy production necessary for neurons to carry out proper synaptic activity, neuronal survival, and differentiation [5, 6]. Furthermore, insulin plays a critical role in the brain and is also involved in the phosphorylation of tau [7, 8], which leads to neurofibrillary tangles, a hallmark of AD-associated neurodegeneration.

The multiple pathophysiological features common to T2DM and AD have paved the way for exploring the beneficial effect of antidiabetic drugs on dementia treatment. Several studies were performed to evaluate the effects of several antidiabetic drugs like incretins, thiazolidines, metformin, and intranasal insulin in the treatment of AD [9]. Surprisingly, incretins showed the most promising neuroprotective effect [10], decreasing neuronal apoptosis, oxidative stress, beta-amyloid accumulation, and tau-induced neurofibrillary tangle formation [11]. Furthermore, several clinical studies demonstrated that glucagon-like peptide-1 receptor agonists (GLP-1RAs) can reduce the risk of dementia in T2DM patients by improving memory and learning and overcoming cognitive impairment [12]. Recently, a dual glucose-dependent insulinotropic polypeptide (GIP) and glucagon-like peptide-1 (GLP-1) receptor agonist, Tirzepatide (TIR), constituting of 39 amino acids, was synthesized [13]. TIR decreases the glycated hemoglobin level in T2DM patients more efficiently than the GLP-1 RA semaglutide [14, 15], can significantly improve systemic insulin sensitivity [16], and is very effective in overcoming insulin resistance [17].

Surprisingly, both GIP and GLP-1 receptors are expressed in the central nervous system (CNS) and seem to play a role in neural progenitor cell proliferation and behavior modification [18–21]. GIP receptors (GIPRs) have been detected in many areas of the brain, including the hippocampus and hippocampal progenitor cells [19, 22, 23]. Hippocampus, Purkinje cells, amygdala, substantia nigra, striatum, and several areas of the thalamus, hypothalamus, and brainstem are all positive for GIP mRNA and/or protein [19, 20, 24]. Thus, it is predicted that a dual agonist of both the incretin receptors might be more efficient in neuroprotection, than a single incretin agonist. Indeed, although GIP and GLP-1 share many common actions in the pancreas, they have been shown to have distinct actions outside of the pancreas [18], and the potential role of TIR on neuroprotection has never been investigated. More recently, a potential protective effect of TIR against spatial learning and memory impairment has been very recently demonstrated in diabetic mice [25] but no data on the molecular processes involved were provided.

So far, the effects of the dual GIP/GLP-1 receptor agonist, TIR, on markers of neuronal growth (CREB and BDNF levels), apoptosis (BAX/Bcl2 ratio) differentiation, (pAkt, MAP2, GAP43, and AGBL4) and insulin resistance (GLUT1, GLUT4, GLUT3 and SORBS1) in human neuroblastoma cell line (SHSY5Y) exposed to normal (NG) and high concentration of glucose (HG) for 7 days were investigated. Due to the well-known high glucose-mediated epigenetic changes, the potential role on DNA methylation of genes involved in neuroprotection and epigenetic modulators of neuronal growth (miRNA 34a), apoptosis (miRNA 212) and differentiation (miRNA 29c) was also investigated.

## Materials and methods

### Cell culture

The SHSY5Y human neuroblastoma cell line was purchased from DSMZ Cell Dive (ACC-209). Cell line has been tested and authenticated following the manufacturer's instruction and it was negative for mycoplasma contamination. The cell line was maintained in an incubator at 37 °C, 95% O_2_, and 5% CO_2_. The cells were grown in DMEM (Microgem cat# AL007) was used along with 15% fetal bovine serum (FBS, Euroclone cat# ECS0180L), 1% l-glutamine (Euroclone cat# ECB3000D), 1% penicillin–streptomycin (Euroclone cat# ECB3001D) and 1% non-essential amino acid (Microgem cat# ACL006). The cells were exposed to normal (NG) (25 mM) and high glucose (HG) (150 mM) [15] (Merck-KGaA, cat# G8644-100ML) for 7 days. Before selecting the appropriate concentration of TIR and initiating experiments, a comprehensive toxicity assessment using cell viability assays and cell counts were performed. Cells exposed to TIR concentrations ranging from 0 µM to 0.4 µM for 7 days exhibited no statistically significant differences in both cell viability percentage and cell numbers (Additional file 1: Fig. S1). Both NG and HG cells, after reaching 70–80% of confluence, were treated with Tirzepatide (Selleckchem, LY3298176 cat# P1206) at concentration of 0.2 μM, since the lower effective dose used in other cell line [26] was ineffective in our setting. Experiments were executed and repeated at least for 3 times. In all experiments, every 48 h cell culture media was replaced.

### Cell proliferation detection

Cell proliferation was assayed by Ki-67 (Alexa Fluor® 488 conjugate) antibody (Cell Signaling cat#11882). According to the manufacturer’s instructions, the cells after 7 days treatment are detached and washed with PBS. The cells were then fixed with 4% formaldehyde followed by incubation for 15 min at room temperature. After washing with PBS, cells were permeabilized using 0.1% PBS-tween on ice for 15 min. According to datasheet 1 × 10^6^ cells were incubated with the antibody for 1 h at room temperature after washing with PBS. Before measurement the cells were again washed 2 times and resuspended in PBS. The measurements were carried out using BD Accuri C6 PLUS Personal Flow Cytometer (BD biosciences) and data processing was performed by FlowJo BD Accuri C6 Plus software for windows [27].

### Protein extraction and Western blot analysis

Cells were dissolved in lysis buffer containing protease inhibitors (Tris–HCl, pH 8, 10 mM; NaCl 150 mM; NaF 10 mM; NP40 1%; PMSF 1 mM). Then, the proteins were subjected to 8% or 10% sodium dodecyl sulphate–polyacrylamide gel electrophoresis (SDS-PAGE) and transferred to 0.22 μm polyvinylidene fluoride membranes. The membranes were blocked with 5% nonfat milk in TBS-T (Tris-buffered pH8/0.15% Tween 20) at room temperature for 1 h and then incubated with primary antibodies diluted in TBS-T (dilutions in according to data sheet), BDNF (1:1000) (Elabscience cat#e-ab-18244), CREB (1:1000) (Cell Signaling cat#9197), SORBS1(1:500) (Atlas antibodies cat# hpa027559), GAP43 (1:1000) (Abcam cat#ab16053), MAP2 (1:500) (Abcam cat#ab32454), GLUT4 (1:1000) (Abcam cat#ab33780), Bcl-2 (1:1000) (Elabscience cat#e-ab-15522), BAX (1:1000) (Abcam cat#ab32503), GLUT1 (1:1000) (Elabscience cat# e-ab-31556), GLUT3 (1:400) (Abcam cat#ab15311), AGBL4 (1:1000) (Gene Tex cat#n2c3), pAkt (1:1000) (Cell Signaling cat#4060) Akt (Cell Signaling cat# 2920) for overnight at 4 °C. For normalization of protein expression Actin (1:1000) (Abcam cat# ab8227) and Vinculin (1:10,000) (Abcam cat#ab129002) were used as internal control. After three washes in TBS-T, the membranes were incubated with corresponding secondary antibodies, with goat anti-rabbit IgG-h + HRP conjugated (Bethyl cat# A120-101p) and donkey anti-mouse IgG-h-I HRP conjugated (Bethyl cat# A90-137p) (1:5000) secondary antibodies, for 1 h at room temperature. Immunocomplexes were visualized by using Clarity Max Western ECL Substrate (Bio-Rad Laboratories cat#1705062) Immunocomplexes were observed through ChemiDOC Imaging System with Image LAB Software (Bio-Rad Laboratories, Version 6.1). The molecular weight of proteins was estimated with pre-stained protein markers (Opti-Protein-Marker abm cat# G623). Densitometry analysis was performed using ImageJ software.

### RNA extraction and miRNA detection

For the isolation and purification of total RNA, including small RNAs from SHSY5Y cells, in the first step, trypsin was used to detach the cells from the flask and collected in 1.5 ml tubes. Then, Trizol and bromocloropropane were added followed by vortexing for 15 s. The tubes were incubated for 15 min at room temperature. After incubation, they were centrifuged for 15 min at 12,000 rpm at 4 °C. The aqueous phase was collected and in which same quantity of chilled isopropanol was added, followed by vortexing. After incubating for more than 1 h at − 80 °C the tubes were again centrifuged for 30 min at 12,000 rpm at 4 °C. The supernatant was discarded, and the pallet was washed with 75% ethanol by centrifugation at 7500 rpm for 10 min. The pallet was resuspended with sterile water and detection of purity and concentration of the RNA were carried out through QIAexpert spectrophotometer (Qiagen cat#1038703). The complementary DNA (cDNA) was synthesized from 2 ng of the total purified RNA by TaqMan MicroRNA Reverse Transcription kit (Applied Biosystem Lithuania cat# 4366597) using specified RT primer (Applied Biosystem cat# 4427975 U6 sn RNA, cat# mir34, cat# mir212 cat# mir29c) following protocol of manufacturer. Rotor-gene Q (Qiagen cat# R0515102) was used to measure the expression of miRNA through utilizing the PrimeTime gene expression master mix (IDT cat#1055772) and the TM primers (Applied Biosystem cat#4427975 U6 snRNA, cat#4440887 mir34a-5p, mir212-3p, mir29c-5p). The PCR condition was polymerase activation at 95 °C for 3 min, followed by 40 cycles of amplification with denaturation at 95 °C for 5 s and annealing at 60 °C for 30 s. All the samples were run in duplicates. For each amplification cycle particular threshold cycle (C_t_) value was obtained and the difference between C_t_ values of targeted miRNAs and U6 were used to calculate ΔC_t_. Then to obtain ΔΔC_t_ difference between the ΔC_t_ of NG group and drug treated group were taken. Finally, for the analysis of fold change 2^−ΔΔCt^ was calculated and its average from three individual experiments was plotted in histogram [28].

### Real time PCR

cDNA was synthesized from 1 μg of total RNA isolated from SHSY5Y cells using QuantiTect Reverse Transcription Kit (Qiagen cat# 205310). mRNA levels were determined by qPCR with SsoAdvanced Universal SYBR Green Supermix (Bio-Rad cat#1725270) using Rotor-Gene Q (Qiagen).

Primer’s sequence:

BDNF

Primer 1: 5′-GCTCTGTGCGATTTCATTGTG-3′

Primer 2: 5′-GCCTTCATGCAACCAAAGTATG-3′

GLUT3

Primer 1: 5′-GAAGACTTGAATTAGATTACAGCGATG-3′

Primer 2: 5′-GAAAGAGCCGATTGTAGCAA-3′

GLUT1

Primer 1: 5′-GTGCCATACTCATGACCATCG-3′

Primer 2: 5′-GGCCACAAAGCCAAAGATG-3′

GLUT4

Primer 1: 5′-TCCAACAGATAGGCTCCGAA-3′

Primer 2: 5′-CCCAATGTTGTACCCAAACTG-3′

BAX

Primer 1: 5′-CAAACTGGTGCTCAAGGC-3′

Primer 2: 5′-AAAGATGGTCACGGTCCAAC-3′

BCL2

Primer 1: 5′-GATGACTGAGTACCTGAACCG-3′

Primer 2: 5′-AGCCAGGAGAAATCAAACAGAG-3′

CREB

Primer 1: 5′-GTCCAAACAGTTCAGTCTTCCT-3′

Primer 2: 5′-GTTACACTATCCACTGACTCCTG-3′

AGBL4

Primer 1: 5′-CTTGTAAGCCAGCACCCTAT-3′

Primer 2: 5′-CAGCCAGTGACGATTCAGAT-3′

SORBS1

Primer 1: 5′-ATCTTCCCACCACCTTAAACC-3′

Primer 2: 5′-GAACCACCATCACATTCAGAAC-3′

MAP2

Primer 1: 5′-TGAAGAACATCCGCCACAG-3′

Primer 2: 5′-ATCTTGACATTACCACCTCCAG-3′

GAP43

Primer 1: 5′-AGCCAAGCTGAAGAGAACATAG-3′

Primer 2: 5′-TTCTTAGAGTTCAGGCATGTTCT-3′

β-ACTIN

Primer: 5′-CATCCGCAAAGACCTGTACG-3′

Primer: 5′-CCTGCTTGCTGATCCACATC-3′

All samples were run in duplicate. For each amplification cycle, a threshold cycle (C_t_) value was obtained, and ΔC_t_ was calculated as the C_t_ difference between target mRNA and housekeeping mRNA (β-Actin). Fold increase of mRNA expression compared with NG was calculated using 2^−ΔΔCt^ method.

### DNA extraction and methylation analysis

The DNA was isolated with the help of QIAamp DNA Blood Mini kit (Qiagen cat#51104) following the protocols of manufacturer. 350 ug of DNA was taken for bisulfite conversion through EpiTect Fast DNA Methylation Kit (Qiagen cat#59824) according to the instructions from manufacturer. In the next step, this bisulfite converted DNA was amplified using Pyromark PCR kit (Qiagen cat#978703) where the PCR conditions were 1 cycle for 15 min at 95 °C and next 40 cycles for 30 s at 94 °C, 30 s at 56 °C and 10 min at 72 °C. For assurance the PCR products were run in 2% agarose gel electrophoresis (Amersham Bioscience). Then the biotinylate PCR products were investigated for pyrosequencing-based methylation utilizing PyroMark Q48 Advanced CpG Reagent (Qiagen cat# 974022) and commercially designed primers including SORBS1_03 (PM00042168) Chromosome 10, bp 97319534–97321622, AGBL4_03 (PM00094297) Chromosome 1, bp 50488739–50490934, CREB 1 (CRTC1_01) (PM00188804) Chromosome 19, bp 18793114–18795233 and BDNF_01 (PM00155491) Chromosome 11, bp 27721460–27723528. For the analysis PyroMark CpG SW 1.0 software (Qiagen) was used [29].

### Pathway enrichment analysis

QIAGEN Ingenuity Pathway Analysis (IPA) software (QIAGEN, Milan, Italy) was used for enrichment analysis. The ‘‘core analysis’’ function was used to interpret the data based on biological processes, canonical pathways, and gene networks. Each gene identifier was mapped to its corresponding gene object in the Ingenuity Pathway Knowledge Base (IPKB). The p-value of 0.05 was set as the cutoff value for the enrichment. The top five enrichment results of the Molecular and Cellular Function were used as a focus point to connect all the available data using the tools “Connect” and “Path Explorer”.

### Statistical analysis

The data were analysed by SPSS IBM Version 23 or GraphPad Prism 7.0 software and expressed as the means ± SEM. Differences between the mean values were considered significant at a p-value of < 0.05. GraphPad Prism software was used for drawing figures.

## Results

### Tirzepatide’s effects on cell proliferation, growth and apoptosis markers in neuronal cells exposed to HG

The effects of TIR on proliferation, and main markers involved in neuronal growth and death were first investigated in SHSY5Y exposed to normal (NG) and high glucose (HG) concentration.

**Fig. 1:**
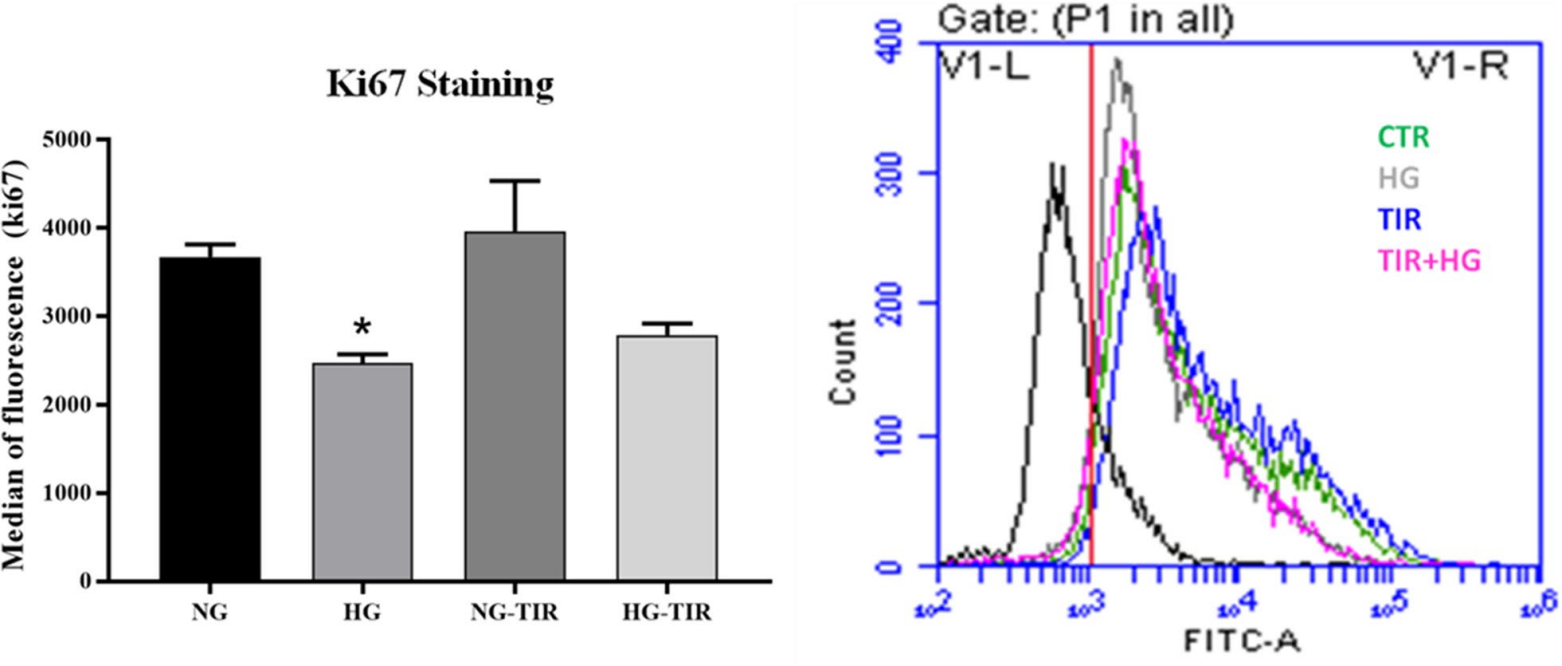
Tirzepatide has no effect on cell proliferation: Cell proliferation analysis was performed through staining cells with Ki67 conjugated to Alexa Fluor® 488. The histogram (left panel) represents median of fluorescence (FITC) and the graph (right panel) is representative image of flow cytometry analysis. The histogram shows the analysis of 3 separate experiments. Data are mean ± SEM. *p < vs NG

The cell proliferation was detected by measuring Ki-67. Cells exposed to HG showed significant Ki-67 median of fluorescence reduction compared to NG (p < 0.05). However, TIR treatment did not show a significant effect on cell proliferation independently of glucose concentration (Fig. 1).

**Fig. 2:**
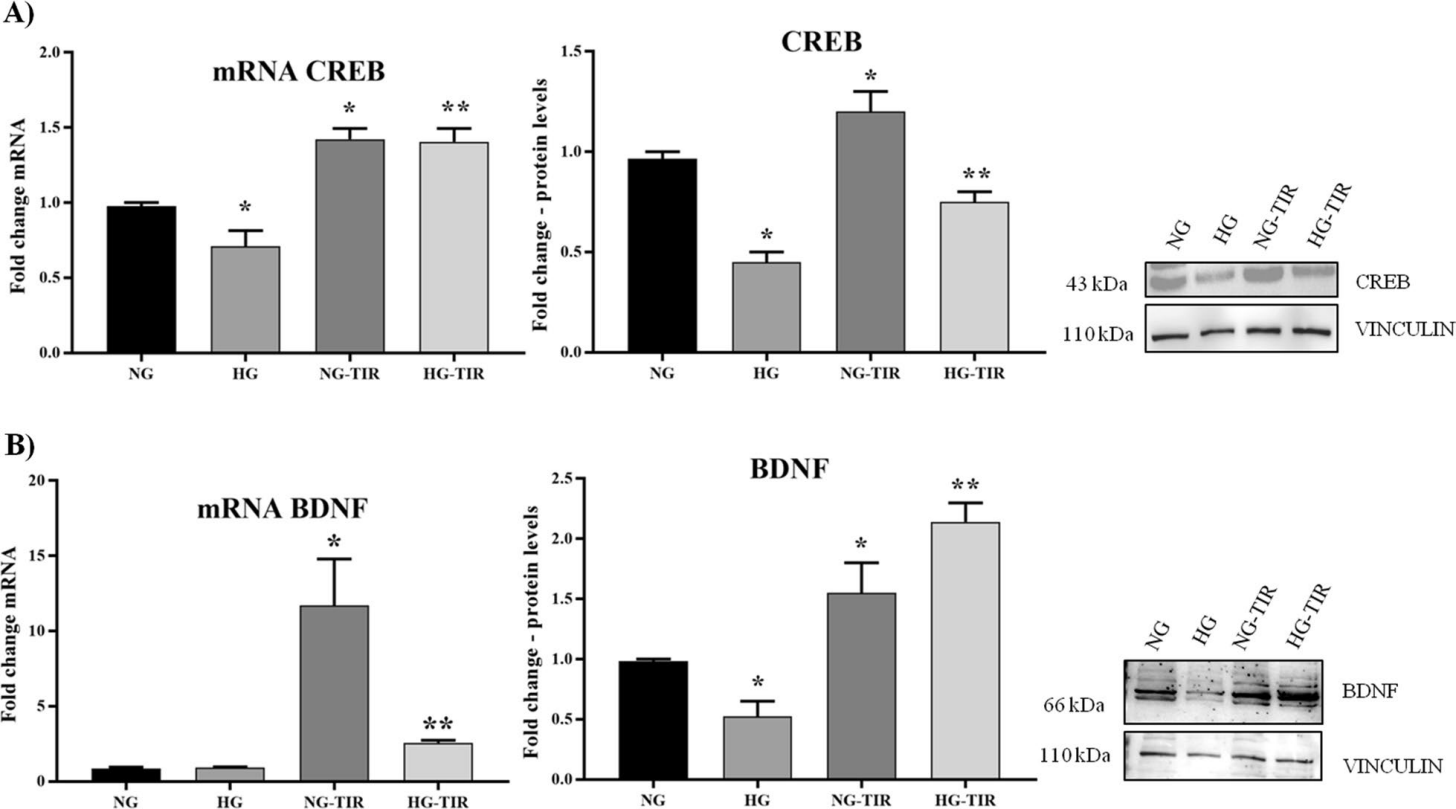
Tirzepatide increases the growth markers expression in neuronal cells exposed to HG: **A** CREB and **B** BDNF mRNA expression levels in SHSY5Y cells (left panel). The fold increase of mRNA expression compared with NG was calculated using the 2^−ΔΔCt^ method. Western Blot analysis of CREB (**A**) and BDNF (**B**) (right panel). The histograms show the densitometric analysis of 3 separate experiments representing the relative expression being NG value set as 1. Data are mean ± SEM. *p < vs NG; **p < vs HG

Moreover, TIR induced upregulation of mRNA expression and protein levels of both CREB and BDNF (Fig. 2A, B) compared to non-treated cells (NG) (p < 0.05 vs NG). HG induced downregulation of both CREB mRNA and protein levels compared to NG cells (p < 0.05) while TIR prevented such HG mediated effect (p < 0.05 vs HG) (Fig. 2A). No statistically significant differences between HG and NG were observed in BDNF mRNA level while HG induced a BDNF protein downregulation (p < 0.05 vs NG) (Fig. 2B). In contrast, TIR upregulated BDNF mRNA expression and prevented the HG-induced protein levels down-regulation (p < 0.05 vs HG) (Fig. 2B).

**Fig. 3:**
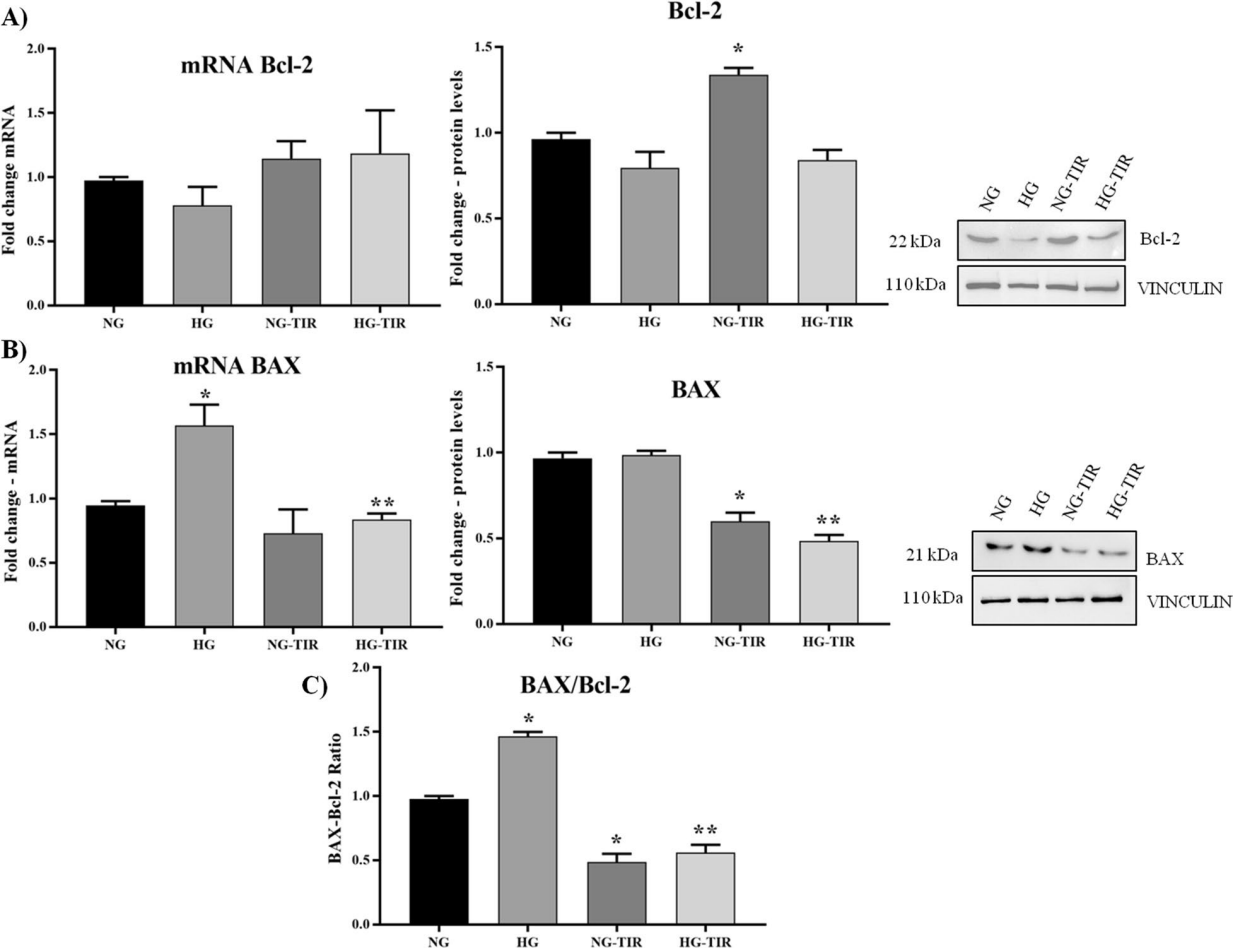
Tirzepatide induces downregulation of apoptosis markers in neuronal cells: **A** Bcl-2 and **B** BAX mRNA expression levels in SHSY5Y cells (left panel). The fold increase of mRNA expression compared with NG was calculated using the 2^−ΔΔCt^ method. Western Blot analysis of Bcl-2 (**A**) and BAX (**B**) (right panel). The histograms show the densitometric analysis of 3 separate experiments representing the relative expression being NG value set as 1. **C** BAX/Bcl-2 protein expression ratio. Data are mean ± SEM. *p < vs NG; **p < vs HG

In addition, TIR treatment (NG-TIR) induced a statistically significant increase in Bcl-2 and decreased BAX protein levels, while no changes in mRNA expression were evidenced. Neither HG nor TIR-HG had any significant effect on Bcl-2 mRNA and protein expression (Fig. 3A).

HG increased BAX mRNA expression, but not protein levels (p < 0.05 vs NG). Addition of TIR (TIR-HG) prevented the mRNA increase and induced a significant decrease of BAX protein levels (Fig. 3B). So far, the protein ratio BAX/Bcl-2, an important apoptotic marker, peaked in presence of HG (p < 0.05 vs NG) but presence of TIR in medium smoothed both NG and HG effect (Fig. 3C).

### Tirzepatide upregulates the expression of neurodifferentiation markers

**Fig. 4:**
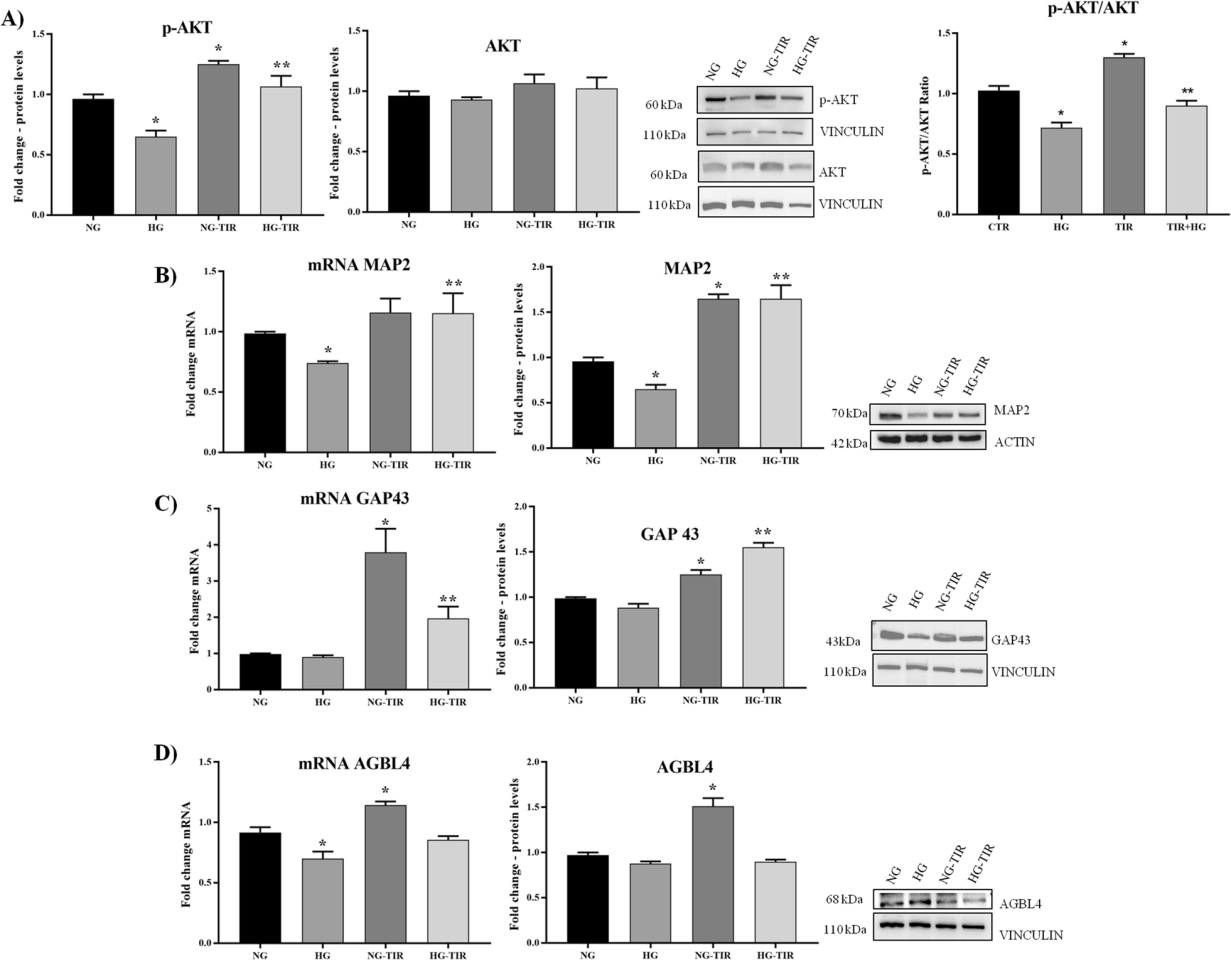
Tirzepatide upregulates the expression of neurodifferentiation markers: **A** Western Blot analysis of AKT (left panel) and p-AKT (central panel). Ratio between the phosphorylated and total forms of AKT (each one normalized with its vinculin), **B** MAP2, **C** GAP43 and **D** AGBL4 mRNA expression levels in SHSY5Y cells (left panel). The fold increase of mRNA expression compared with NG was calculated using the 2^−ΔΔCt^ method. Western Blot analysis of MAP2 (**B**), GAP43 (**C**) and AGBL4 (**D**) (right panel). The histograms show the densitometric analysis of 3 separate experiments representing the relative expression being NG value set as 1. Data are mean ± SEM. *p < vs NG; **p < vs HG

Cell differentiation was evaluated by analyzing the main neurodifferentiation markers, such as pAkt, MAP2, GAP43, and AGBL4 (Fig. 4).

Along with NG, TIR treatment induced a statistically significant increase in pAkt, MAP2, GAP43, and AGBL4 protein levels (Fig. 4A–D, right panel). Nevertheless, only for GAP43 and AGBL4, we observed a parallel significant increase in mRNA and protein levels (Fig. 4C, and D, left panel). HG vs NG showed a significant decrease in pAkt and MAP2 protein levels (Fig. 4A, and B, right panel) and a decrease in MAP2 and AGBL4 mRNA expression (Fig. 4B, and D left panel). TIR-HG prevented the HG-induced changes in protein levels of pAkt, MAP2, GAP43 (Fig. 4A–C, right panel) and MAP2 and GAP43 mRNA expression (Fig. 4B and C, left panel). In cells exposed to HG, TIR failed to affect AGBL4 mRNA and protein levels (Fig. 4D).

### Tirzepatide induces upregulation of main glucose transporters in neuronal cells

**Fig. 5:**
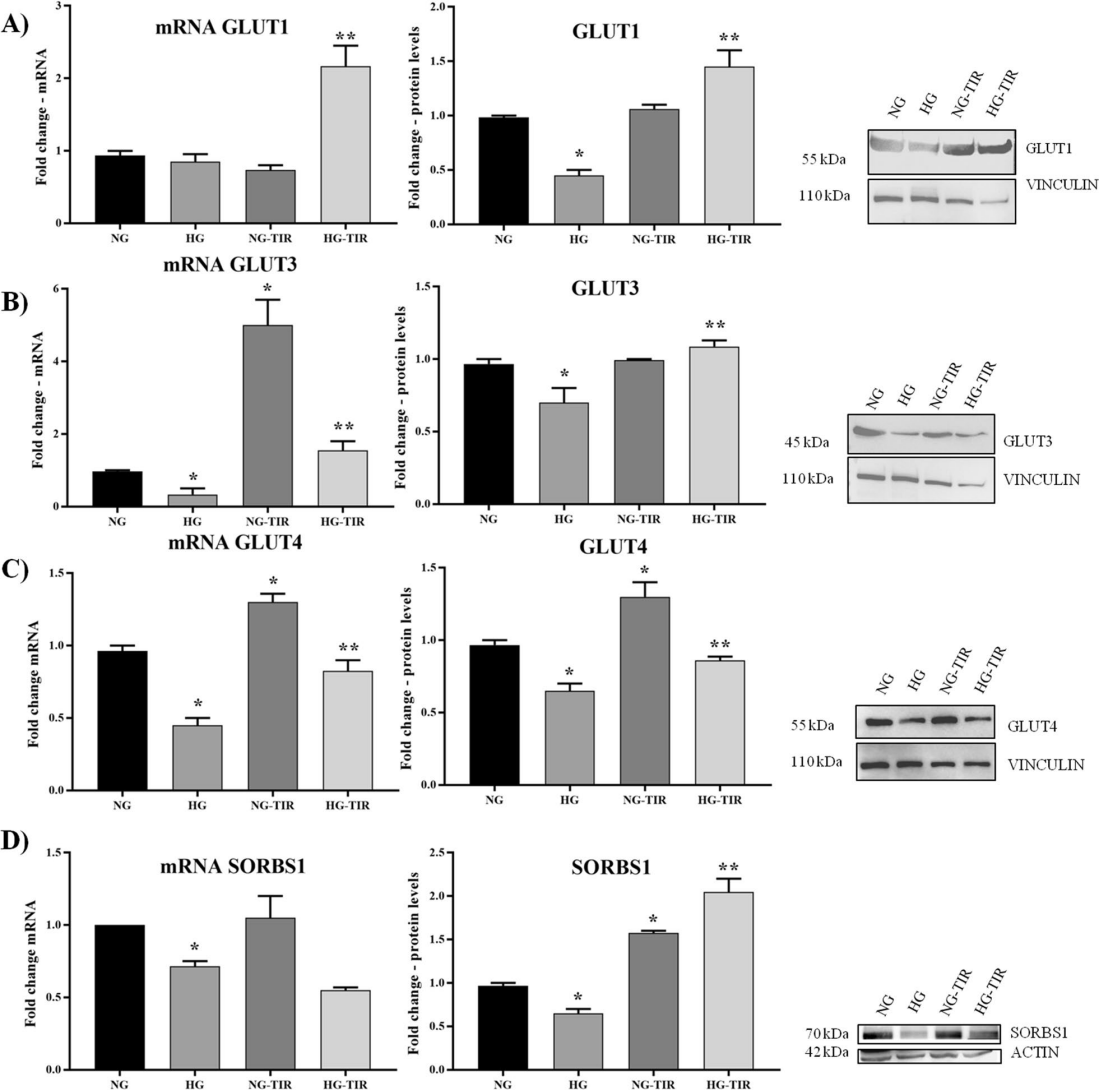
Tirzepatide induces upregulation of main glucose transporters in neuronal cells: **A** GLUT1, **B** GLUT3, **C** GLUT4, and **D** SORBS1 mRNA expression levels in SHSY5Y cells (left panel). The fold increase of mRNA expression compared with NG was calculated using the 2^−ΔΔCt^ method. Western Blot analysis of GLUT1 (**A**), GLUT3 (**B**), GLUT4 (**C**) and SORBS1 (**D**) (right panel). The histograms show the densitometric analysis of 3 separate experiments representing the relative expression being NG value set as 1. Data are mean ± SEM. *p < vs NG; **p < vs HG

To better understand the TIR role on neuronal glucose metabolism, the mRNA and protein levels of GLUT1, GLUT3 and GLUT4 and SORBS1 [30, 31], a major regulator of insulin-stimulated signaling and glucose uptake, were investigated (Fig. 5). TIR treatment induced a statistically significant increase in GLUT3 and GLUT4 mRNA expression (Fig. 5B and C, left panel) and in GLUT4 and SORBS1 protein levels [32] (Fig. 5C and D, right panel).

HG vs. NG showed a significant decrease in GLUT3 and GLUT4, and SORBS1 mRNAs Fig. 5B–D, left panel) and a significant decrease in GLUT1, GLUT3, GLUT4, and SORBS1 proteins (Fig. 5A–D, right panel). Treatment with TIR (HG-TIR) prevented HG-induced changes observed in mRNA expression and protein of glucose transporters and insulin sensitivity marker levels.

### Tirzepatide regulation of CREB, BDNF, SORBS1 and AGBL4 DNA methylation and miR-34a, miR-212 and miR-29c expression

**Fig. 6:**
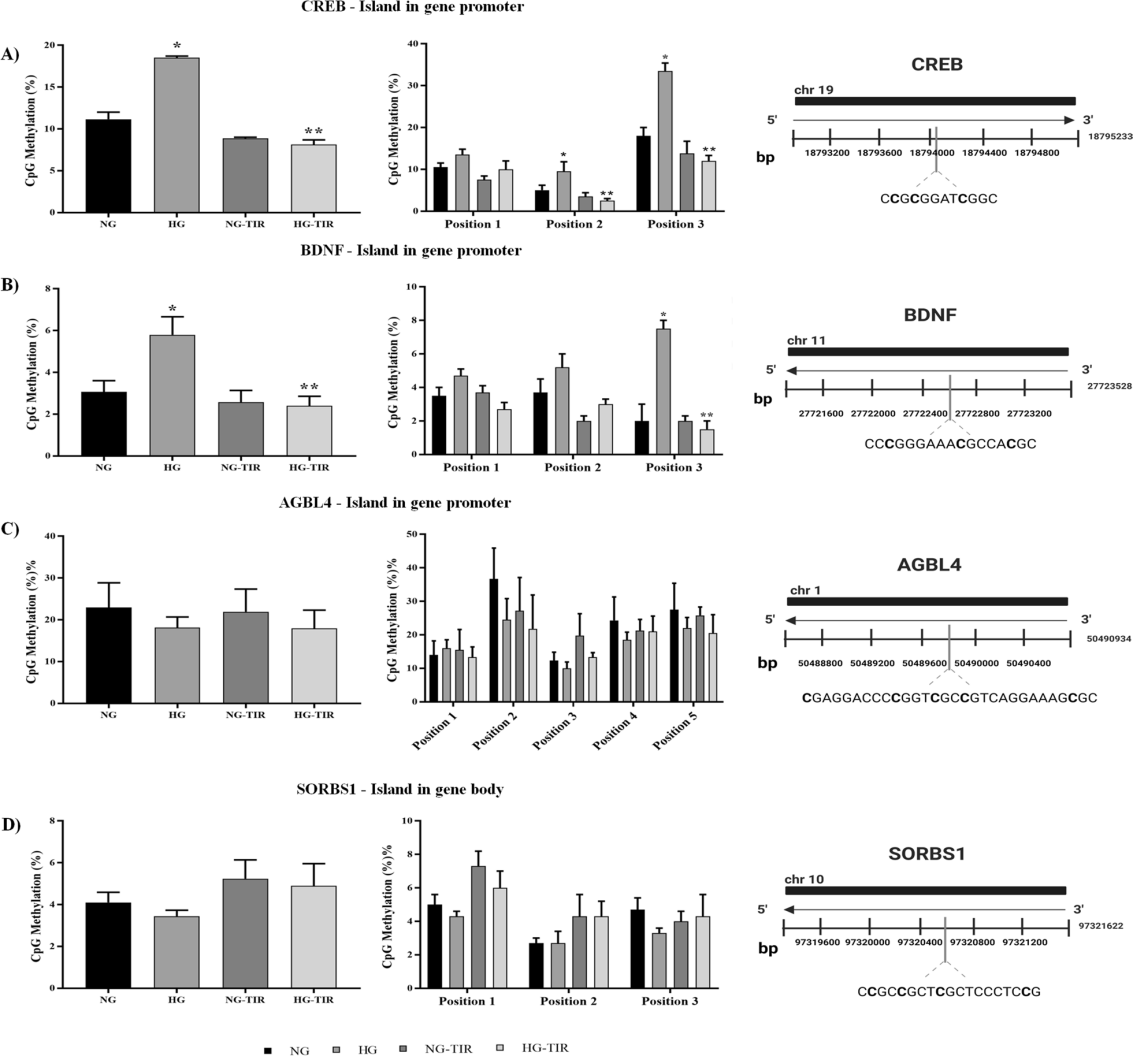
Tirzepatide regulates the DNA methylation of CREB, BDNF, SORBS1and AGBL4 genes in neuronal cells treated with HG: **A**–**D** DNA methylation analysis expressed as percentage of CpGs methylation and schematic representation of CpG site locations in human CREB, BDNF, AGBL4 promoters and SORBS1 gene body. Data are mean ± SEM. *p < vs NG; **p < vs HG

TIR did not affect DNA methylation in all genes investigated along with NG incubation. HG vs NG showed a statistically significant increase in total DNA methylation levels in the promoter region of CREB and BDNF (Fig. 6A, and B). HG-induced a statistically significant increase in methylation levels in positions 2 and 3 of CREB (p < 0.05 vs NG) and in position 3 of BDNF genes (Fig. 6A, and B, right panel). Such HG related changes were prevented by TIR addition (Fig. 5A and B).

No effect of HG and HG-TIR treatment on DNA methylation of AGBL4 promoter regions and SORBS1 gene body (Fig. 5C, and D) was observed.

**Fig. 7:**
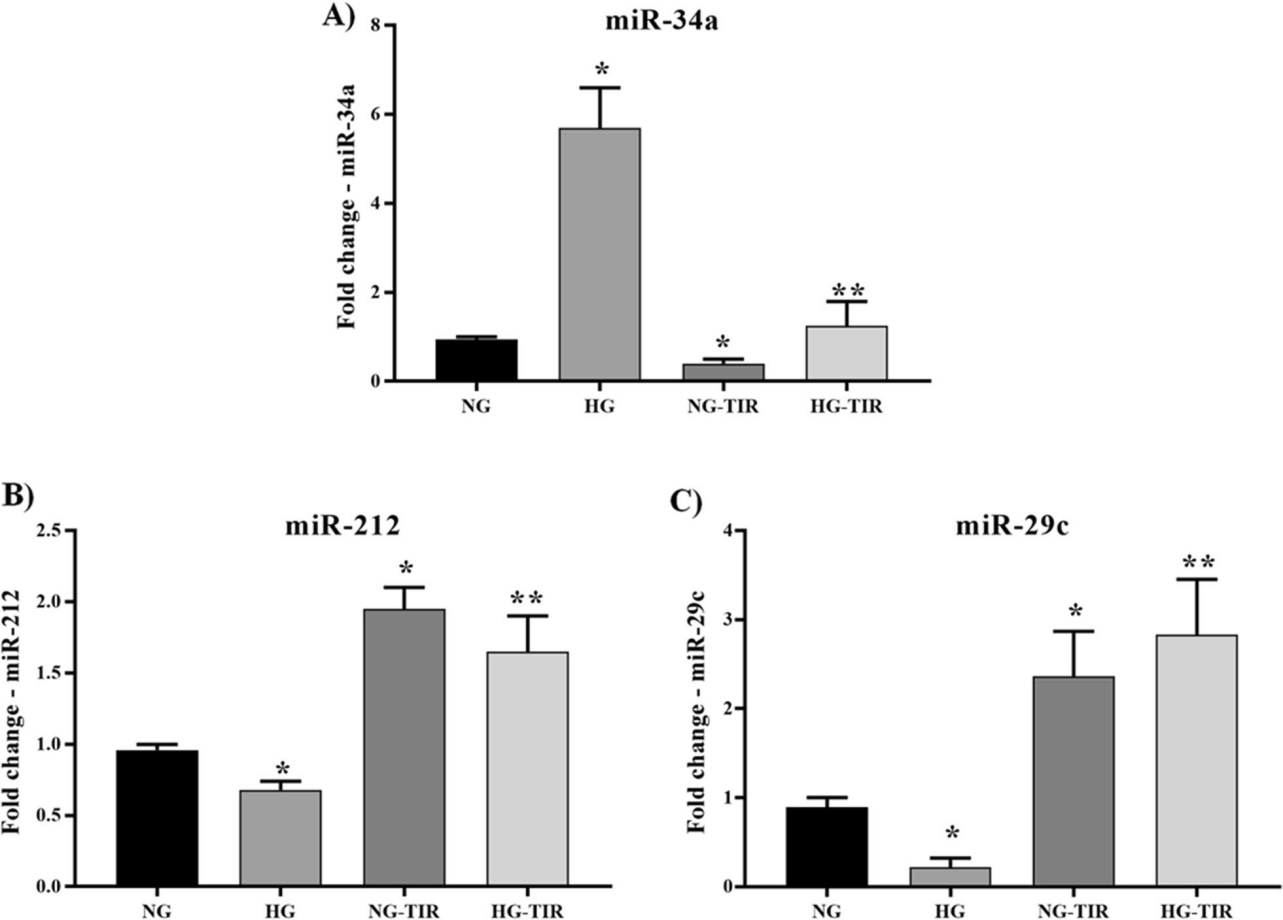
Tirezepatide regulates the miRNA expression: miR-34a, miR-212 and miR-29c. **A** miRNA-34a, **B** miRNA-212 and **C** miRNA-29c expression analysis in SHSY5Y. U6 was used as internal control. The fold increase of miRNA expression compared with NG was calculated using the 2^−ΔΔCt^ method. Data are mean ± SEM. *p < vs NG; **p < vs HG

TIR treatment vs NG induced a statistically significant decrease in miR-34a and an increase in miR-212 and miR-29c (Fig. 7A–C). HG showed a significant increase in miR-34a and a significant decrease in miR-212 and miR-29c expression levels. All the HG-related changes were reversed by TIR addition.

### Corroborating "in silico" analysis of Tirzepatide effects

**Fig. 8:**
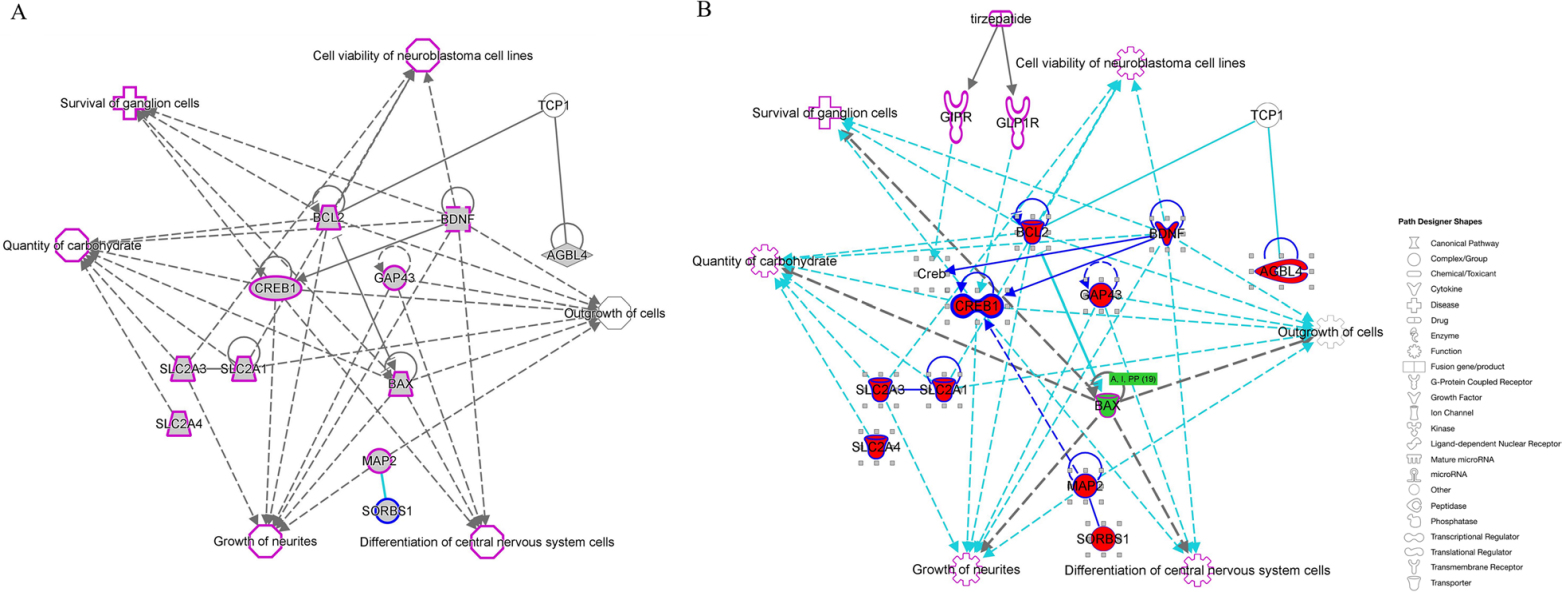
Building interactive models of experimental systems **A** without and **B** with Tirzepatide. The key entries genes of this work are labeled in larger bold font with red fill. Direct connections between/among genes are shown in solid lines; indirect interactions are shown as dashed lines (also called “edges”). Connections between genes objective of this work are shown in dark blue; interactions between highlighted genes and not directly mapped in this work are shown in turquoise. Target shapes are indicative of function, and the complete legend has been reported in right part of figure

The interactive model confirmed the connections among analyzed markers and the main pathways in which they are involved (Additional file 1: Table S1). These analysis highlighted connections between already available targets in a dataset and uncovers relevant relationships between genes of interest enabling the identification of the shortest paths between molecules. To further explore the potential role of TIR, subnetworks, and canonical pathways were interrogated by adding to the interactive model TIR. The simulation showed and confirmed that TIR (Fig. 8) interfered with the main networks and pathways that were found activated in SHSY5Y human neuroblastoma cell line.

## Discussion

Our study firstly demonstrates that, TIR can affect main molecular pathways involved in neuronal growth (CREB/BDNF), anti-apoptotic process (BAX/Bcl-2), neuro-differentiation (pAkt, MAP2, GAP43 and AGBL4) and neuronal glucose homeostasis (GLUT1, GLUT3 and GLUT4). A role in regulating DNA methylation of genes involved in neuroprotection and epigenetic modulators of neuronal growth (miRNA 34a), apoptosis (miRNA 212), and differentiation (miRNA 29c) was also observed.

Several evidences emphasized the relationship between hyperglycemia and neurodegeneration due to their cross-linking molecular pathways [33] and the effectiveness of antidiabetic drugs like incretins, thiazolidines, and metformin on preventing neurodegeneration. Among them, incretins showed the most promising neuroprotective effect [9] reducing the risk of dementia in T2DM patients by improving memory, and learning, and overcoming cognitive impairment [12]. A novel drug, TIR has been recently synthesized, able to bind both GLP-1 and GIP incretin receptors and with a stronger effect in decreasing glycated hemoglobin and in improving systemic insulin sensitivity in T2DM, when compared to the GLP-1RA [14, 17]. So far, a potential protective effect against spatial learning and memory impairment has been, only recently, addressed in diabetic mice [25] but, the molecular processes involved remain unknown.

Our results firstly demonstrated that 7 days of TIR treatment significantly activated the CREB/BDNF signaling cascade and the associated downstream pathways in neuroblastoma cell line (SHSY5Y) exposed to normal (NG) and high glucose (HG) concentration thus suggesting potential implications of TIR in term of neuroprotection and neurodegenerative disorders prevention.

TIR induced an upregulation of protein levels of CREB and BDNF compared to no treated cells (NG) and prevented the pAkt/CREB/BDNF downregulation induced by HG. Interestingly enough, treatment with TIR also prevented the hypermethylation induced by HG (p < 0.05 vs HG) in both CREB and BDNF genes promoter and induced a remarkable reduction in miR-34a expression, a regulator of CREB transcription whose overexpression was observed in aged and neurodegenerative brain, resulting in impaired synaptic plasticity and cognitive performance [34, 35].

CREB, a member of a large functionally- and structurally related group of transcription factors, has a critical role in nervous system growth and development, synaptic plasticity, long-term memory formation and in the neuroprotective response to pathophysiological effectors [35–38]. The molecular mechanisms by which CREB regulates neuronal survival are centered on the transcription of BDNF, which belongs to a family of neurotrophins that have a crucial role in the survival and differentiation of neurons during development [39]. The neurotrophins are also involved in plasticity changes related to learning and memory. In the adult brain, BDNF also maintains high expression levels and regulates both excitatory and inhibitory synaptic transmission and activity-dependent plasticity [40, 41]; in contrast, BDNF deficiency promotes AD progression, dis-homeostasis, neurohormone defects, and the accumulation of neurotoxins [42].

Recent studies indicate that CREB protects neurons regulating the anti-apoptotic gene B Cell Lymphoma-2 (Bcl-2) expression [43]. An increase in BAX/Bcl-2 ratio has been shown to result in memory loss and learning capacity deregulation in the cortex and hippocampus region [44]. Additionally, the phosphorylation of Akt has been associated with the activation of neuronal differentiation markers like microtubule-associated protein-2 (MAP2) growth-associated protein-43 (GAP-43) and the ATP/GTP binding protein-like 4 (AGBL4) [45–48]. These proteins are important targets for treating AD-related tauopathies [46]. In particular, MAP-2 is a cytoskeletal phosphoprotein mainly associated with microtubules and postsynaptic densities that affect neuronal plasticity; GAP-43 is a presynaptic secreted protein highly expressed during neuronal development and synaptogenesis in the hippocampus and the related cortices [49–52]; AGBL4 takes part in neuronal differentiation by promoting axonal growth and axonal transport of mitochondrion.

Interestingly enough, in our experimental model, TIR treatment was associated with a significant decrease of an important apoptotic prognostic marker, BAX/Bcl2 ratio, and with MAP2, GAP-43 and AGBL4 upregulation in cells exposed to both normal and HG levels. Furthermore, an upregulation of miR-212 and miR-29c, both involved in the regulation of the expression of the mentioned genes, was also observed in both normal and high glucose conditions. miR-212 is transcriptionally regulated by CREB [53] and its overexpression leads to an increase in Bcl-2 and a reduction in BAX level [54] and miR-29c directly targets Akt signaling pathway, thereby serving as a neuroprotector. In our cell model, both miRNAs were reduced after 7 days of HG. Thus, the observed effects of TIR on miRNA might partly explain its role in regulating studied molecular pathways. Indeed, the mechanism of action of miRNAs can affect either the transcription (mRNA level) or translation (protein level) [55] and justify the different trends observed between mRNA and protein levels of some markers analyzed.

According to the experimental evidence that apoptosis is the main mechanism for neuronal death in neurodegenerative diseases and that all the investigated proteins are important targets for treating AD-related abnormalities, our results support the role of TIR as an intriguing therapeutic possibility for the treatment of neurodegenerative disorders beyond its hypoglycemic effects.

Finally, several studies demonstrated that memory processing and cognitive functions depend on neuronal glucose metabolism and sensitivity [56] and that glucose homeostasis disruption is one of the mechanisms by which T2DM patients develop AD [57–59]. Moreover, hyperglycemia by modulating the expression of glucose transporters induces neuronal insulin resistance [60]. In the brains of AD patients, a significant reduction in the expression of GLUT1, GLUT3, and GLUT4 was observed, impacting neuronal activity [61], progressive neuronal loss, and neurodegeneration [58, 62]. Intriguingly, in our study, we observed that TIR treatment prevented the HG induced mRNA and protein downregulation of GLUT3 along with GLUT1, GLUT4, and SORBS1, a protein that takes part in diabetes pathogenesis [31], and regulates GLUT4 translocation [32] affecting neuronal insulin resistance [63].

Our results agree with previous data showing that TIR significantly activated PI3K/AKT/GSK3β signaling pathway ameliorating insulin resistance in the hippocampus of diabetic rats [25].

Our results not only confirm the potential role of TIR in the activation of the pAkt/CREB/BDNF downstream signaling cascade but also support its role in neuroprotection and in the prevention of the deleterious effect of hyperglycemia and insulin resistance on the mentioned pathways. Furthermore, the effects observed on CREB and BDNF promoter methylation and in regulating miR-34a, miR-212, and miRNA-29c levels, highlighted the role of TIR in overcoming the epigenetic modifications induced by hyperglycemia.

## Conclusions

Our results firstly demonstrate the potential role of TIR in ameliorating high glucose-induced neurodegeneration and overcoming neuronal insulin resistance by targeting molecular and epigenetic modulators of neuronal growth, apoptosis, differentiation, and survival. Further specifically designed clinical studies will be necessary for validating the “in vitro” results and supporting the potential clinical implications of TIR treatment in terms of neuroprotection and neurodegenerative disorders prevention.

### Supplementary Information

**Additional file 1.** **Supplementary Figure 1**: Effects of different concentrations of Tirzepatide on cell survival rate percentage and cell count; **Table 1**: The enriched pathways depicted in **Figure 8**; Representative images of the whole-length blots

## Data Availability

The datasets used and/or analysed during the current study are available from the corresponding author on reasonable request.

## Abbreviations

AD: Alzheimer's disease
AGBL4: ATP/GTP binding protein like 4
BAX: Bcl-2-like protein 4
Bcl2: B-cell lymphoma 2
BDNF: Brain-derived neurotrophic factor
CNS: Central nervous system
CREB: CAMP response element-binding protein
DMEM: Dulbecco's modified eagle medium
FBS: Fetal bovine serum
GAP43: Growth associated protein 43
GIP: Glucose-dependent insulinotropic polypeptide
GLP1-RAs: Glucagon-like peptide 1 receptor agonists
GLUT1: Glucose transporter 1
GLUT3: Glucose transporter 3
GLUT4: Glucose transporter 4
HG: High glucose
Ki-67: Antigen Kiel 67
MAP2: Microtubule-associated protein 2
NG: Normal glucose
pAkt: Phospho protein kinase B
PBS: Phosphate-buffered saline
SHSY5Y: Neuroblastoma cell line
SORBS1: Sorbin and SH3 containing domain
T2DM: Type 2 diabetes mellitus
TIR: Tirzepatide
Tris–HCl: Tris hydrochloride
NaCl: Sodium chloride
NaF: Sodium fluoride
PMSF: Phenylmethylsulfonyl fluoride
SDS-PAGE: Sodium dodecyl sulphate–polyacrylamide gel electrophoresis
TBS: Tris-buffered saline
