# Supplementary material for: Tirzepatide prevents neurodegeneration through multiple molecular pathways
Source: J Transl Med. 2024 Jan 29;22:114. doi: 10.1186/s12967-024-04927-z (PMC10823712; doi:10.1186/s12967-024-04927-z)
Supplement: Supplementary file 1 — Additional file 1. Supplementary Figure 1: Effects of different concentrations of Tirzepatide on cell survival rate percentage and cell count; Table 1: The enriched pathways depicted in Figure 8; Representative images of the whole-length blots [file 12967_2024_4927_MOESM1_ESM.docx]

**Tirzepatide prevents neurodegeneration through multiple molecular pathways**

Rosaria Anna Fontanella^1§^, Puja Ghosh^1§^, Ada Pesapane^1^, Fatemeh Taktaz^1^, Armando Puocci^1^, Martina Franzese^1^, Maria Federica Feliciano^1^, Giovanni Tortorella^1^, Lucia Scisciola^1*^, Eduardo Sommella^2^, Concetta Ambrosino^3,4^, Giuseppe Paolisso^1, 5^, Michelangela Barbieri^1^

***^1^*** *Department of Advanced Medical and Surgical Sciences, University of Campania "Luigi Vanvitelli", Naples, Italy.*

*^2^Department of Pharmacy, University of Salerno, Fisciano, SA, Italy*

***^3^*** *Biogem Institute of Molecular Biology and Genetics, Ariano Irpino, Italy.*

***^4^*** *Department of Science and Technology, University of Sannio, Benevento, Italy.*

**^5^***UniCamillus, International Medical University, Rome – Italy*

^§^*The authors share the co-first authorship*

***Corresponding author:**

Dr. Lucia Scisciola

Department of Advanced Medical and Surgical Science, University of Campania “Luigi Vanvitelli”, Napoli, (Italy)

e-mail: lucia.scisciola@unicampania.it

**Supplementary Material - Extended Figures and Table**

**Additional Figure S1**

**
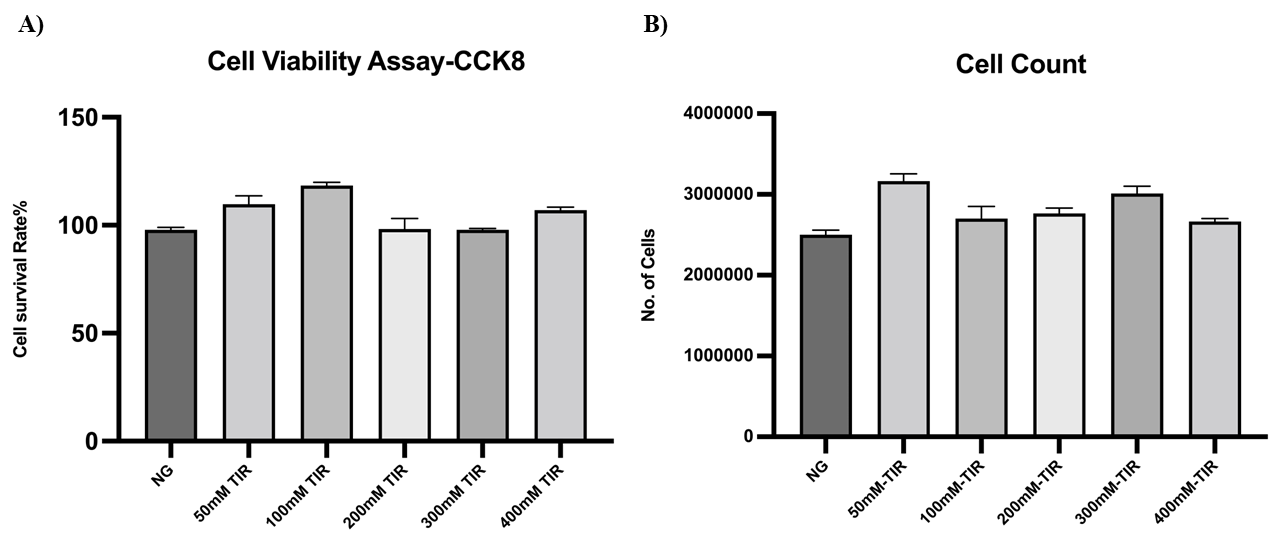
**

**Figure S1: Effects of different concentrations of Tirzepatide on cell survival rate percentage and cell count.:**

Cell viability was assayed by Cell Counting Kit-8 (CCK-8, CK04, Dojindo) according to the manufacturer's protocols. Briefly, SHSY5Y cells were seeded into 96-well plates and treated with Tirzepatide for 7 days. At the end of 7 days, the cell counts were taken. After specific treatment, 10 μL of CCK-8 solution was added to each well and incubated for 2 h at 37°C. The absorbance was then recorded at 450 nm using a microplate reader (Infinite M Nano^+^ plate reader, TECAN). The relative cell viability was normalized with the control group using optical density values, and three independent experiments were conducted.

**Table S1**

**Additional file - Representative images of the whole-length blots**

**Figure S2**


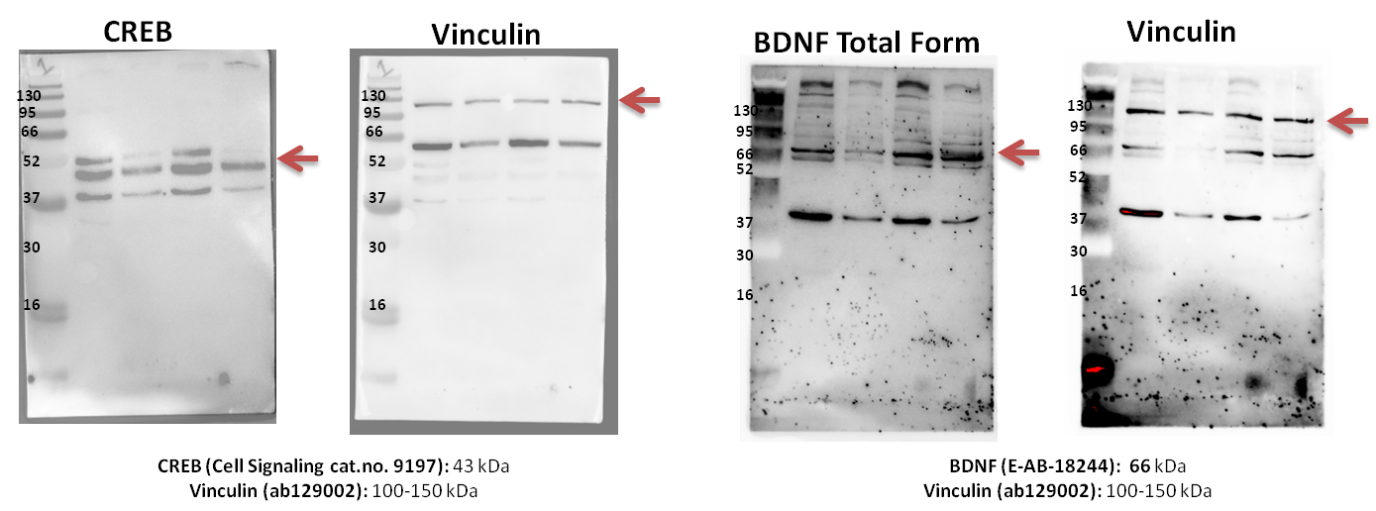


**Figure S3**


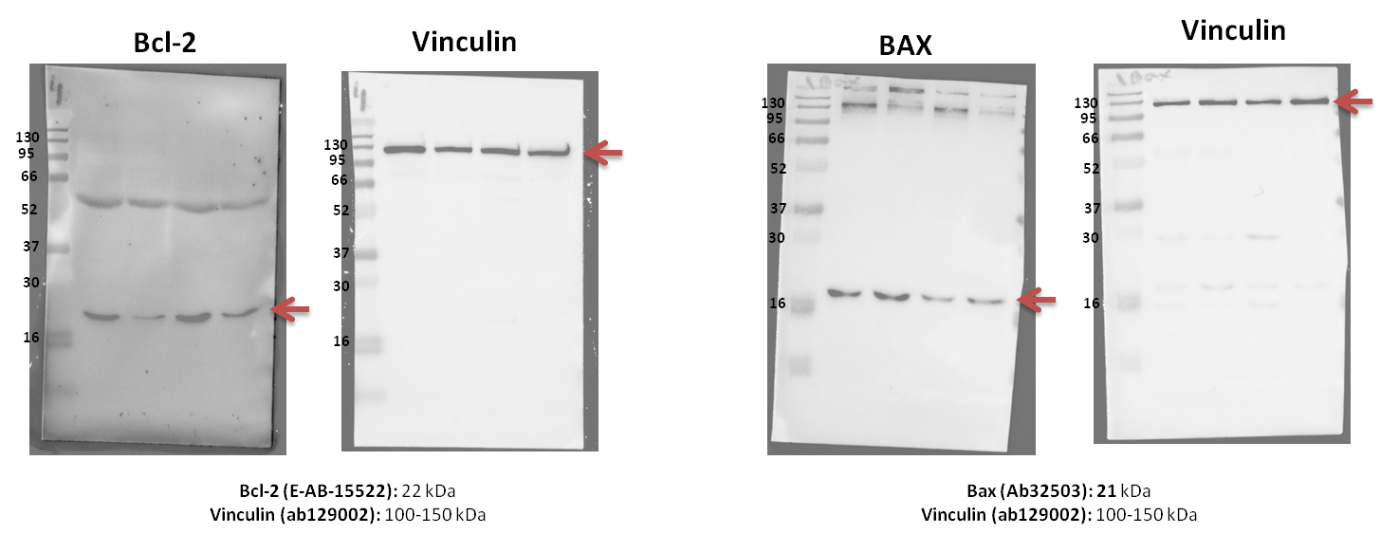


**Figure S4
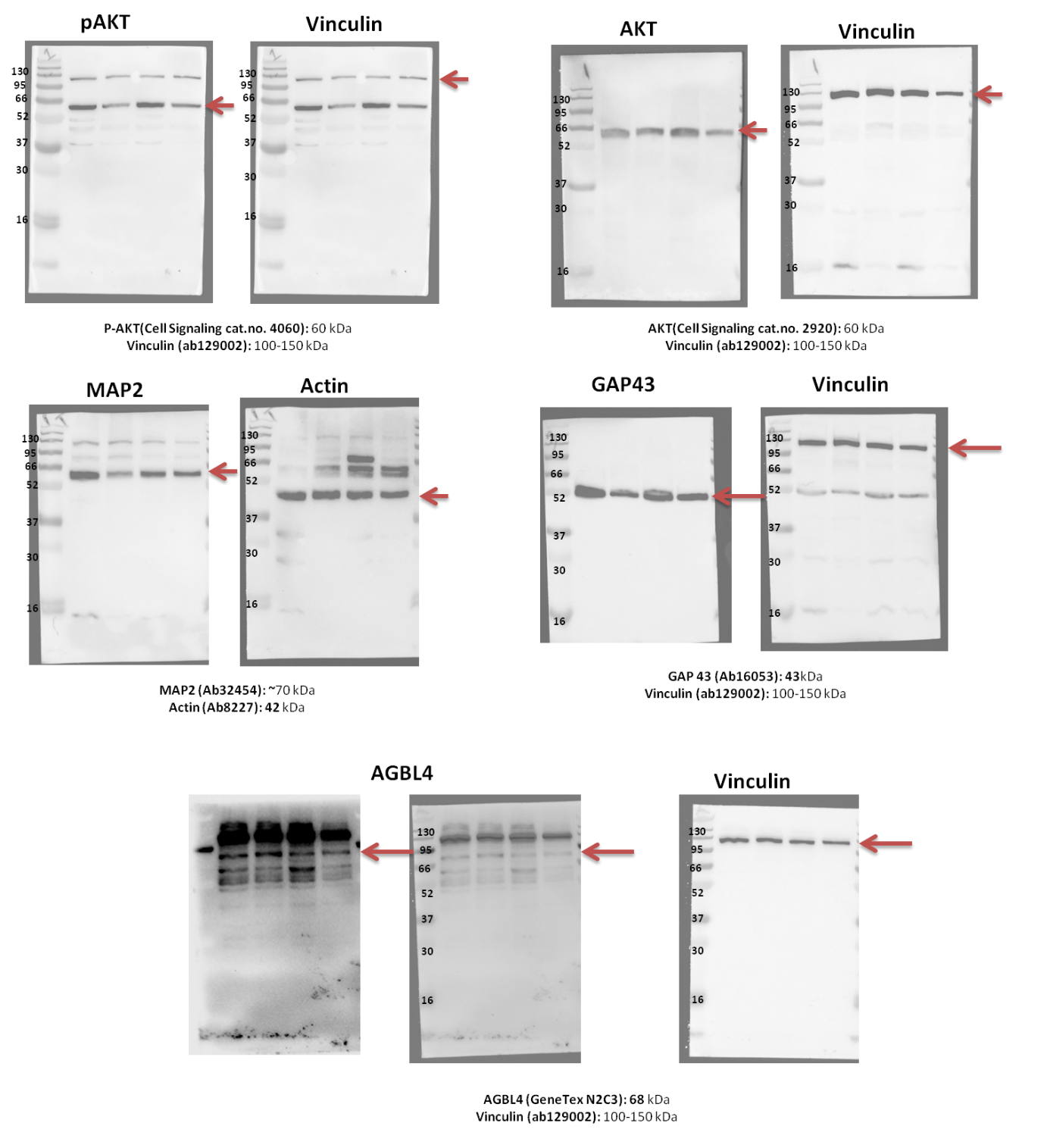
**

**Figure S5
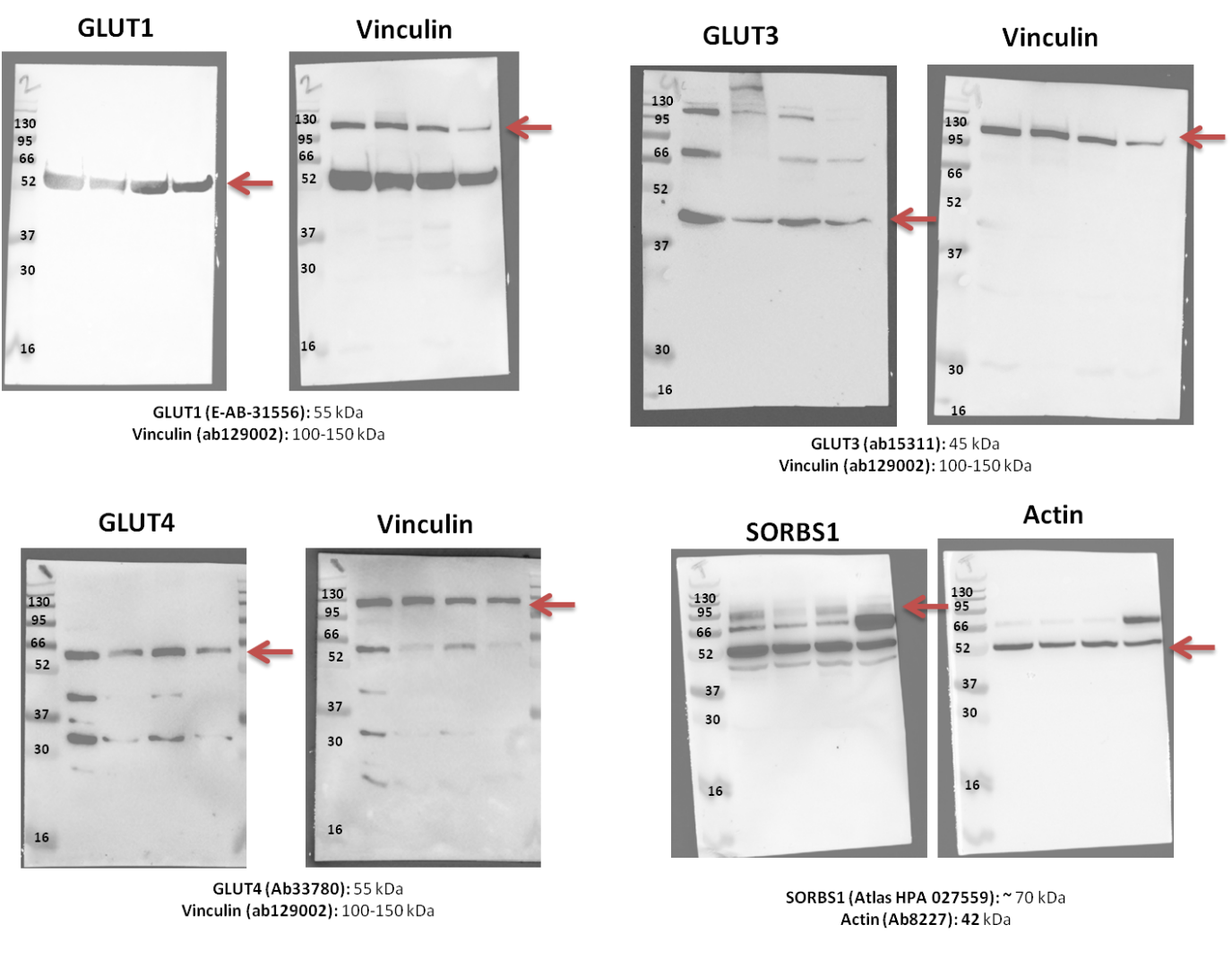
**
